# Influence of Post-Processing on the Properties of Multi-Material Parts Obtained by Material Projection AM

**DOI:** 10.3390/polym15092089

**Published:** 2023-04-27

**Authors:** Pablo Zapico, Pablo Rodríguez-González, Pablo Robles-Valero, Ana Isabel Fernández-Abia, Joaquín Barreiro

**Affiliations:** 1 Department of Construction and Manufacturing Engineering, University of Oviedo, Campus of Gijón, 33204 Gijón, Spain; zapicopablo@uniovi.es; 2 Department of Mechanical, Informatics and Aerospace Engineering, University of León—Universidad de León, Campus de Vegazana, 24071 León, Spain; probv@unileon.es (P.R.-V.); aifera@unileon.es (A.I.F.-A.); joaquin.barreiro@unileon.es (J.B.)

**Keywords:** additive manufacturing, multi-material, material projection, post-processing

## Abstract

The great geometric complexity that additive manufacturing allows in parts, together with the possibility of combining several materials in the same part, establishes a new design and manufacturing paradigm. Despite the interest of many leading sectors, the lack of standardization still makes it necessary to carry out characterization work to enjoy these advantages in functional parts. In many of these techniques, the process does not end with the end of the machine cycle, but different post-processing must be carried out to consider the part finished. It has been found that the type of post process applied can have a similar effect on part quality as other further studied process parameters. In this work, the material projection technique was used to manufacture multi-material parts combining resins with different mechanical properties. The influence of different post-processing on the tensile behavior of these parts was analyzed. The results show the detrimental effect of ultrasonic treatment with isopropyl alcohol in the case of the more flexible resin mixtures, being advisable to use ultrasonic with mineral oil or furnace treatment. For more rigid mixtures, the furnace is the best option, although the other post-processing techniques do not significantly deteriorate their performance.

## 1. Introduction

Additive manufacturing technology (AM) is based on part manufacturing by adding layers of material. Originally called rapid prototyping, it was conceived to produce prototypes quickly and easily from digital models. Given its advantages in terms of simplicity of use, great freedom of design and customization without a significant increase in cost, the possibility of reducing the number of components, etc., it is currently of great interest to many sectors such as medical [1], aerospace [2], energy industry [3], sports [4,5], and those geared towards the manufacture of micrometer-scale components [6].

One of the AM techniques that has received great attention in recent times is the Material Projection (MP) technique [7]. This technique allows for the addition of material layers by selective deposition of light-curing resin droplets that are subsequently cured by an ultraviolet light source. For this purpose, AM machines have a head that incorporates piezoelectric injectors arranged in a matrix to deposit the resin, as well as a blade or roller and several ultraviolet light lamps, which flatten and cure the resin once it has been deposited [8].

The main manufacturers of AM machines based on this technique (3D Systems [9] and Stratasys [10]) have developed equipment capable of mixing several light-curable polymer resins simultaneously at the voxel level (3D pixel) to manufacture multi-material parts [11,12]. This new paradigm undoubtedly marks a milestone in terms of the ability to simplify designs by reducing the number of components, due to the variety of properties that these resins can present. The support material used in this technique consists of a paraffin or hydrophobic material that is deposited next to the photopolymers that must be removed by post-processing out of the machine.

The MP technique has proven useful for manufacturing functional parts, in general [13,14], for manufacturing plastic injection molds for short or one-off series [15,16] and for validating prototype parts for series production [17,18]. In addition to the aforementioned advantages of additive manufacturing, this technique has minimal material waste, lower cost, and greater supply chain efficiency compared to machining or small series injection molding [19]. This has made it of great interest to industries as varied as biomedical [20,21] and aeronautics [22].

Nevertheless, MP does not escape the challenges that remain to be addressed for additive manufacturing, among which can be highlighted both the lack of design and manufacturing guidelines, as well as the lack of specific standardization [23]. This has led several researchers to focus in recent years on studying this technique in depth, mainly focusing on the material properties’ characterization [24,25], studying the geometric and surface quality [26,27,28], analyzing the anisotropy of the mechanical properties of the printed parts [29], and manufacturing structures reinforced with multiple materials [30,31].

The interesting results achieved by these works reinforce the need to address the AM challenges mentioned above. Specifically for MP, there is a key issue related to the development of manufacturing procedures: the post-processing operation for removing support material. This operation is mandatory and common to all parts obtained by this technique. As seen in many of the aforementioned works, researchers have focused on analyzing the properties and the economy of the parts depending on their orientation and position in the machine working volume, while avoiding the implications of the needed post-processing. In this respect, a few works mention the problems related to this post-processing. Liu et al. [32] note the advantage of using integrated lattices over snap-fitted lattices in terms of energy absorption but state the difficulty of removing the support material from the former when the structure is very intricate. Something similar is highlighted by Meisel et al. [33] in their work evaluating different limitations of the process, among which stands out the elimination of support material in the case of thin sections. Eren et al. [34] recognize that the removal of support is extremely difficult when printing delicate and thin cell structures, as this limits the minimum cell size and affects the final mechanical response of the reticular structures. Abayazid et al. [31] note that the use of the post-processing recommended by manufacturers can generate warping and geometric distortions of the printed parts. Meanwhile, He et al. [35] note the extreme force that must be applied and its adverse effects on the final quality of the parts in the case of using certain support materials that are different from the usual ones. Therefore, there is a clear need to analyze in depth the influence of post-processing.

On the other hand, it is worth highlighting the few works that analyze the properties or establish design guidelines for using something that is relatively common in this technique, i.e., the combination of different resins with different properties in the same part. This characteristic undoubtedly makes this technique stand out within additive manufacturing due to the simplification of the design in terms of reducing the number of components, as it allows different materials with different properties to be combined in the same part. With regard to the combination of materials, Boopathy et al. [36] studied the energy absorption capacity of printed parts against impact, observing that the different combinations of materials and the direction of the load played an important role in the behavior of the composite material.

This work focused on analyzing two questions that have been little studied or outright overlooked in other works on MP and that are of vital importance to safely exploit the advantages of this AM technique: the good tensile behavior of components that combine several materials with different mechanical properties, as well as the influence that the post-processing to be applied has on this behavior. For that, in the first part of the work, the tensile mechanical behavior of standardized specimens combining a stiff and tough resin with a more flexible one is analyzed. After verifying the good performance of the multi-material specimens, the second part of the work analyzes the influence of different usual post-processing procedures, i.e., using heat or wet ultrasound (with vegetable oil and with isopropanol), on the tensile behavior of multi-material specimens, identifying the most suitable post-processing for the different resin combinations.

## 2. Materials and Methodology

### 2.1. Materials

**Figure 1:**
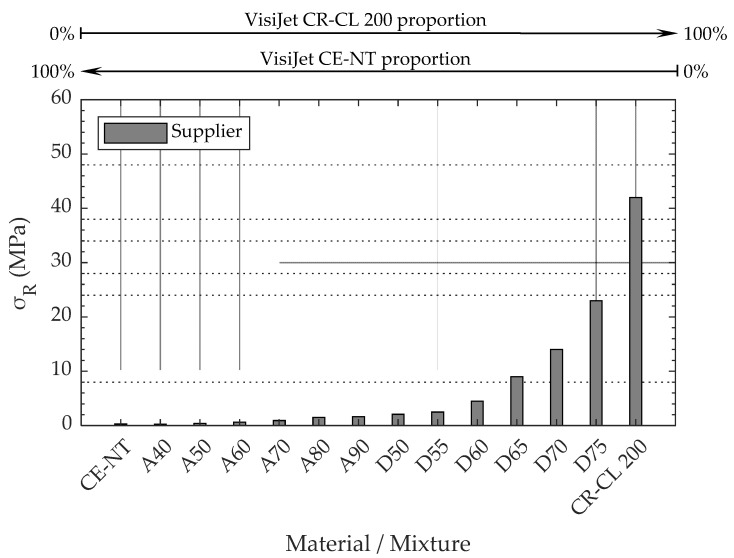
Tensile strength of VisiJet CE-NT, VisiJet CR-CL 200, and the mixtures established in 3D Sprint software [9].

**Figure 2:**
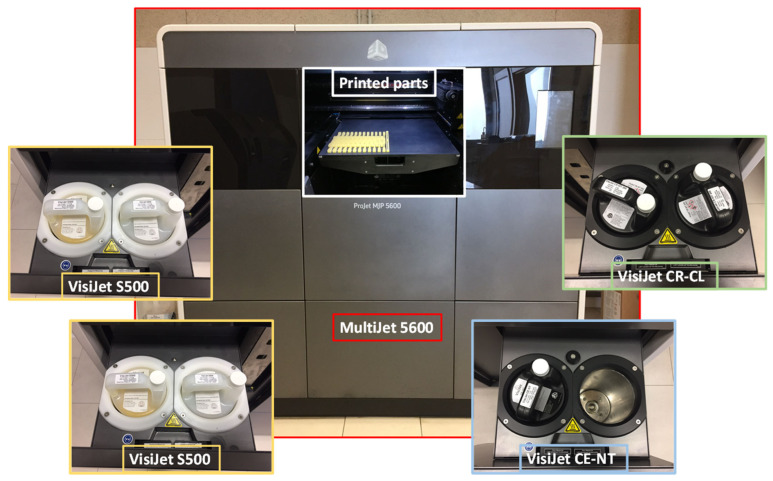
Projet MJP 5600 machine with a set of specimens and VisiJet materials.

The AM Material Projection Machine ProJet^®^ MJP 5600 (3DSystems, Rock Hill, SC, USA) was used in this study. The working volume of this machine is 518 × 381 × 300 mm^3^, with a maximum resolution in the XYZ axes of 750 × 750 × 2000 DPI. As manufacturing material, this machine uses various resins, including mixtures of these in different proportions. The resins used in this work, both supplied by the 3D System (Rock Hill, SC, USA), are VisiJet CR-CL 200 (translucent acrylic based photopolymer resin) and VisiJet CE-NT (amber acrylic-based photopolymer resin). After MP manufacturing, the VisiJet CR-CL 200 resin is comparatively much stiffer and stronger than VisiJet CE-NT, which shows a more flexible behavior. Table 1 shows the tensile strengths of these resins and of the different resin mixtures configurable in the manufacturing software [9]. These tensile strengths are represented in Figure 1, where it can be seen that the higher the proportion of VisiJet CR-CL 200 in the material mixture, the higher the tensile strength achieved. For the pure resins, the supplier also provides the tensile modulus, 0.27–0.43 MPa and 1400–2100 MPa, and the elongation at break, 160–230% and 14–22%, VisiJet CE-NT and VisiJet CR-CL 200, respectively. Figure 2 shows the AM machine used and the carbides of the different VisiJet materials.

As support material, this machine uses VisiJet S500, also supplied by 3D Systems, which is a non-toxic wax. During the MP process, both the resins and the support material are heated to around 60 °C, so that they acquire an appropriate viscosity for projection onto the manufacturing platform. After projection, the resins are cured by ultraviolet light, while the wax is solidified by cooling.

**Figure 3:**
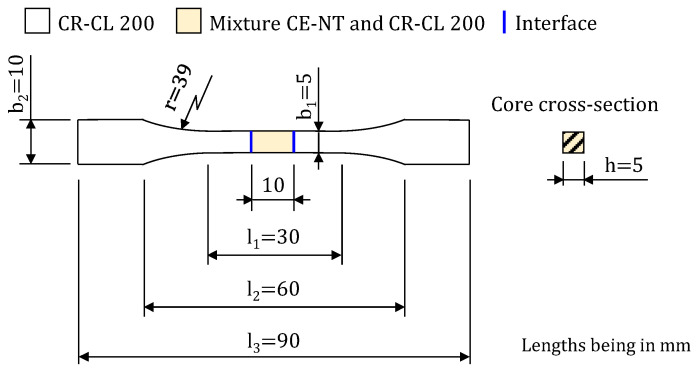
Schematic of the 1BA multi-material specimen designed according to ISO 527-2 [37].

In order to evaluate the tensile mechanical properties of components containing areas made of different resins or resin mixtures, a tensile specimen was designed with a central area which can be made of a different material than that used for the heads. This specimen was designed according to the recommendations of ISO 527-2 [37], with the geometrical parameters represented in Figure 3. As can be seen, the specimen is always manufactured with the strongest material, CR-CL 200, in the head area to ensure the correct gripping of the tensile grips in the tensile test, while in the core, any of the 14 possibilities shown in Table 1 can be selected. Therefore, this specimen not only allows us to determine the tensile behavior of the weakest material used, which is the weakest link in the chain, but also to validate the good behavior of the interface between materials (see Figure 3). All the specimens manufactured were oriented horizontally and with their longitudinal axis perpendicular to the direction of movement of the machine head in order to avoid the main effect of the characteristic anisotropy of this process [38].

Before the parts produced with this machine can be used, it is necessary to remove the VisiJet S500 material in which they are embedded immediately after the MP process. For this purpose, both a DIGITHEAT-TFT furnace (JP Selecta, Barcelona, Spain) and a Digital Pro ultrasonic tank (GT Sonic, Meizhou, China) were used, using either isopropyl alcohol (99.9% purity) or vegetable oil as the fluid in the latter. In some cases which are described in the Methodology section below, the roughness of the central area of the specimens was measured using a Surftest SJ-500 roughness tester (Mitutoyo, Kawasakishi, Japan). The different manufactured specimens were tensile tested using a ME-402 universal machine (Servosis, Madrid, Spain) equipped with a 5 kN load cell. In order to determine the actual dimensions of the center section of the specimens, all of them were measured with a Mitutoyo universal calliper before being tested. During the tests, the length of the central area of the specimen, $l_{1}$, was taken as the reference length for the calculation of the length deformations.

### 2.2. Methodology

**Figure 4:**
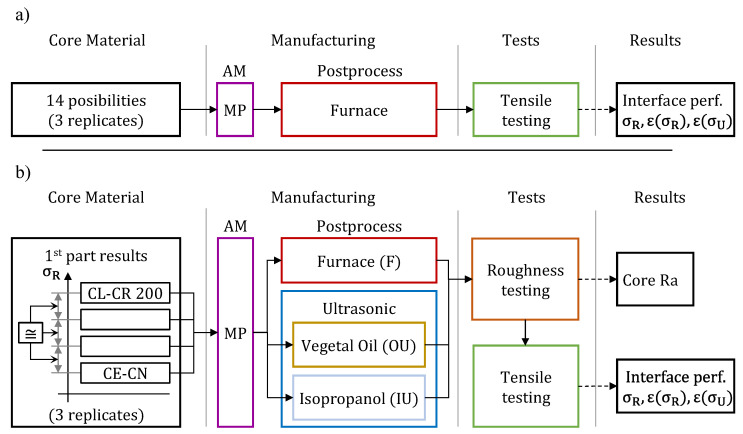
Diagram of procedure followed in the parts of the work: (**a**) first part and (**b**) second part.

This work was carried out in two parts. In the first part (Figure 4a), the tensile strength behavior of specimens combining different resins mixtures was analyzed. These specimens were manufactured using the ultrahigh-definition mode for XY axes, but with a layer height of 16 µm for productivity reasons. For this purpose, 3 specimens were manufactured with each of the possible VisiJet resin combinations (Table 1), equaling 42 specimens in total. After the MP process, these specimens were post-processed to remove the VisiJet S500 material with two 10-min thermal cycles in the furnace at 60 °C. During each of these cycles, the specimens were placed horizontally on a rack, analogous to the manufacturing orientation, so as to avoid possible warping and geometric distortions caused by heat. Between the two furnace cycles, the specimens were removed from the oven for a short period of time, during which the excess wax was manually removed using a piece of absorbent paper. Subsequently, after cooling to room temperature, they were tensile tested according to ISO527-1 recommendations [37], using a speed of 1 mm/min.

**Figure 5:**
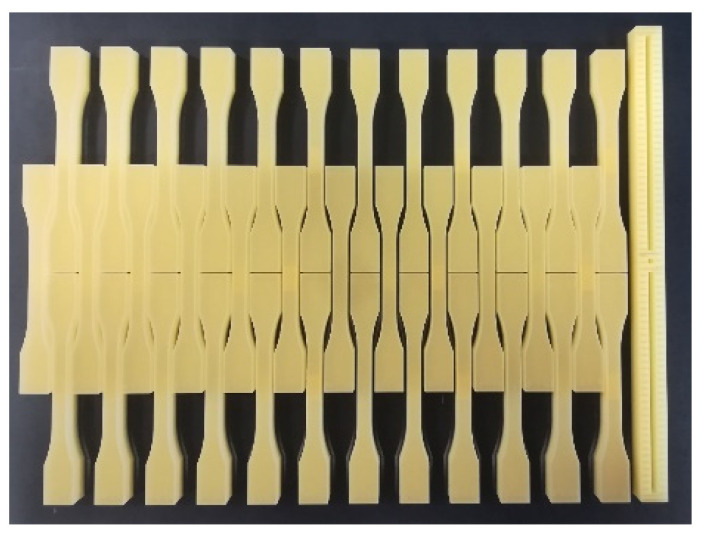
Specimens produced for the second part of the study before post-processing.

Once the good interface performance of the multi-material specimens analyzed in the first part of the work was verified, the influence of the post-processing used on the mechanical properties of these specimens was analyzed in the second part of the work (Figure 4b). In this part, the least and greatest strength specimens, CE-NT and CL-CR 200, respectively, were manufactured, as well as two additional specimens that had shown in the first part tests a strength equi-spaced in magnitude with respect to the previous ones. Three replicates of each of them were manufactured, making thirty-six specimens in total, as shown in Figure 5. These specimens were manufactured with the same machine configuration as the one used in the first part of the work. Then, the replicates were post-processed with each of the following procedures:

- F: two furnace cycles at 60 °C;
- OU: two ultrasonic cycles with mineral oil at 60 °C;
- IU: two ultrasound cycles with isopropyl alcohol at 60 °C.

Each of the above cycles lasted 10 min, and the specimens were manually cleaned with absorbent paper between the two cycles, in each type of post-processing. During the ultrasonic cycles, a 40 kHz frequency was applied when each specimen was completely immersed in the treatment tank, which was filled with the appropriate fluid, i.e., mineral oil or isopropyl alcohol. After post-processing, the roughness of the core of the specimens was measured using a λ_c_ of 2.5 mm and λ_s_ of 8 μm, following the recommendations of the ISO 4288 standard [39], and subsequently a tensile test was performed under the same conditions as in the first part of the work.

To prevent environmental or machine-specific conditions from affecting the results, both the specimens tested in the first part and those tested in the second part were manufactured and tested within 24 h after manufacturing. In addition, the specimens were tested in random order [40]. All specimens were manufactured at the highest machine resolution and with the same material cartridges.

## 3. Results and Discussion

**Figure 6:**
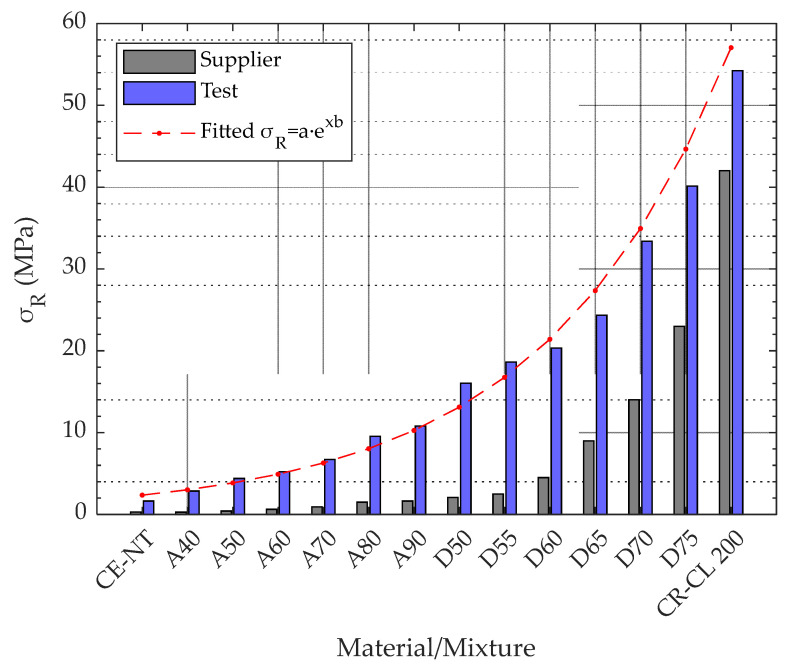
Tensile strength for different material mixtures.

The 42 specimens analyzed in the first part of the work required 14 h of fabrication on the AM machine, consuming 46 g of VisiJet CE-NT, 196 g of VisiJet CR-CL, and 132 g of VisiJet S500. Post-processing took about 2 h. The results of the tensile test carried out on these specimens are shown in Figure 6. As can be seen, it follows a similar exponential behavior to that of the characteristics sheet provided by the manufacturer, but with higher resistances. For specimens of material from CE-NT to D70, the observed strength is several times that specified by the manufacturer, while for the D75 and CR-CL 200 specimens, 74.5% and 29.2% more strength were obtained, respectively.

**Figure 7:**
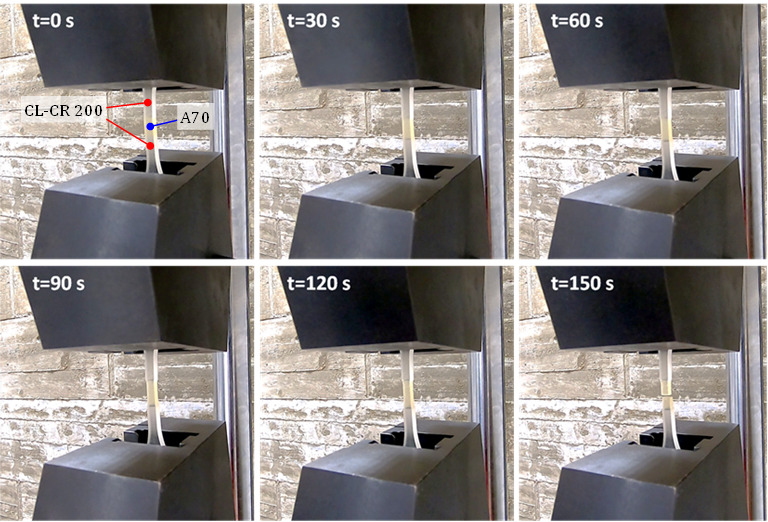
Sequence of the tensile test on specimen A70.

In all cases, the interface between the core material or mixture and the head material, i.e., CR-CL 200, showed adequate behavior, with the rupture occurring in the central section of the core. This can be seen in the sequence of photographs captured during the testing of the A70 core material specimen (Figure 7).

Figure 6 shows the exponential behavior observed in the tensile strength of the specimens as a function of the specimen core composition. It can be approximated by the function represented in Equation (1) with an r-square of 98.02%, where P represents the ratio between CE-NT and CR-CL 200, so that 1 is equivalent to 100% CE-NT and 14 is equivalent to 100% CR-CL 200. This behavior, together with an improvement in the AM machine-control software, would allow more intermediate materials to be defined between the two extremes, so that they could be manufactured with a more suitable strength for each application.

(1) $$\sigma_{R}=1.8475\cdot e^{0.2450\cdot P}\;\mathrm{MPa},\;\mathrm{where}\;P\in \left[1,14\right]$$

**Figure 8:**
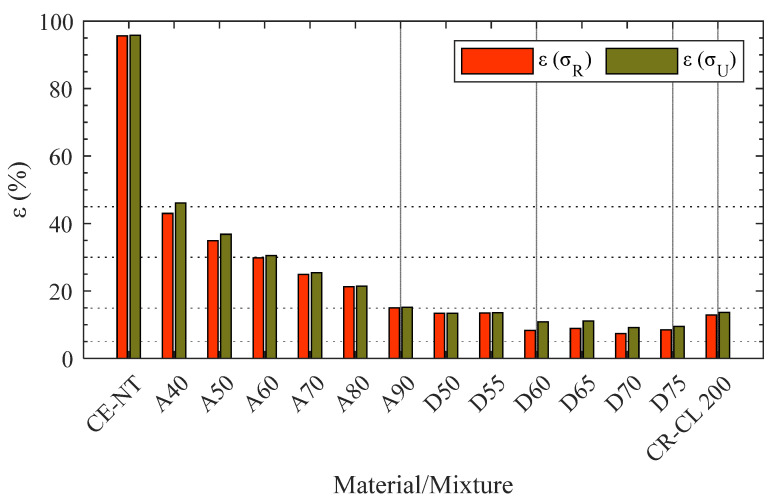
Elongation at tensile strength, $\varepsilon \left(\sigma_{R}\right)$, and at ultimate strength, $\varepsilon \left(\sigma_{U}\right)$, for the different materials and mixtures (reference length, $l_{1}$).

Figure 8 shows the elongation of specimens manufactured with different materials at tensile strength, $\varepsilon (\sigma_{R})$, and at the moment of breaking, i.e., at ultimate strength $\varepsilon (\sigma_{U})$. On the one hand, the similarity of both elongations can be highlighted, as well as the exponential decreasing behavior as a function of the CR-CL 200 material concentration. In the case of the specimen made entirely of this material, CR-CL 200 (in Figure 8), the elongation obtained in the test, i.e., 14%, is within the range indicated by the supplier [9].

After verifying the good performance of the analyzed specimens, in the second part of this work, the roughness of the core area and the tensile behavior as a function of the type of post-processing were studied. This study was performed for those specimens with lower and higher strength and additionally two specimens with equally spaced tensile strength values: CE-NT, D60, D70, and CR-CL 200. From each of these specimens, 3 replicates were manufactured, in total 36 specimens, to be post-processed in three different ways (Figure 4b): F, IU, and OU.

**Figure 9:**
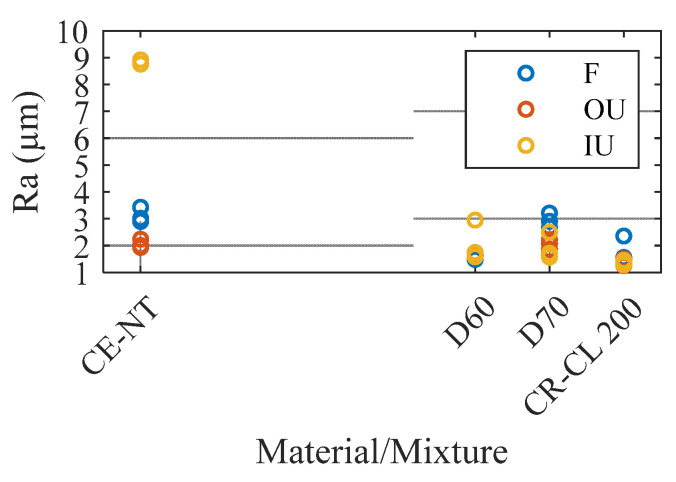
Ra values measured in the core of the specimens.

The Ra roughness values obtained in the core area of the specimens are presented in Table 2 and represented in Figure 9. As can be seen, the specimen whose roughness is most altered depending on the post-processing used is the CE-NT specimen. In this specimen, a much higher roughness is obtained in the case of applying ultrasonic post-processing with isopropanol (IU), 8.87 µm on average in front of 2.03 µm from using mineral oil, and of 3.10 µm obtained with the furnace post-processing. This indicates a chemical interaction between isopropanol and the CE-NT material that alters its surface and, therefore, could also alter its integrity. In the rest of the specimens, no significant variation is observed when changing the post-processing, obtaining Ra values in the range of 2 µm.

**Figure 10:**
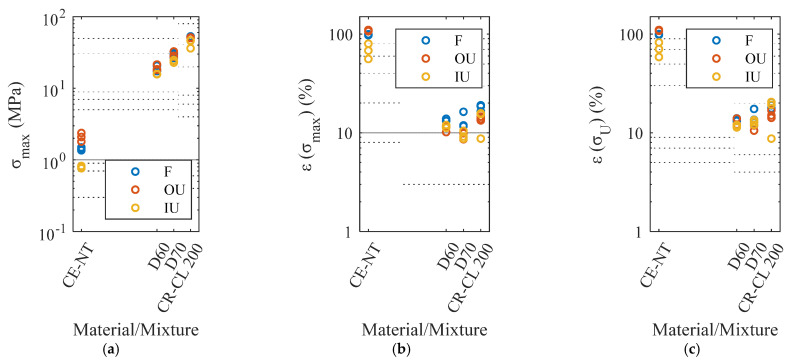
Tensile behavior as a function of post-processing type (F, OU, and IU): (**a**) tensile strength, (**b**) strain at tensile strength, and (**c**) strain at ultimate strength.

The results of tensile strength, strain at tensile strength, and strain at ultimate strength obtained in the tensile tests are shown in Figure 10a–c, respectively. Different statistical models were fitted to analyze these results in search of conclusions about the effect of the different post-processing on that tensile performance. The type of post-processing was used as the input parameter, and the following as output indicators were used: tensile strength, strain at tensile strength, and strain at ultimate strength. The *p*-values shown in Table 3 were obtained from these adjustments.

As can be noticed, the type of post-processing influences the tensile strength reached by the material. Looking at Figure 10a, it can be seen that IU post-processing is the most detrimental. The most affected material is CE-NT with regard to the roughness study (Figure 9). For this material, it is interesting to use the OU post-processing to achieve the best properties, while for the rest of the materials, the F and OU post-processing, are practically equivalent (Figure 10a). In the case of the strain at tensile strength, the IU post-processing should again be avoided for CE-NT, with the effect of the other two processes being similar, while for the D60 and D70 materials, the F post-processing stands out as being beneficial in comparison with the other two (Figure 10b). Finally, regarding elongation at ultimate strength, a correlation was found only for the CE-NT material, with the IU post-processing again being detrimental (Figure 10c).

## 4. Conclusions

In the first part of this work, the tensile mechanical performance of specimens manufactured with Visijet CR-CL 200 material heads and core mixtures of this material in different proportions with Visijet CE-NT was analyzed. These specimens were manufactured using the material projection technique on a ProJet^®^ MJP 5600 machine, whose manufacturer, 3DSystems, also supplies the material. After the design of a 1BA tensile specimen by following the recommendations of the ISO 527-2 standard, 42 of these specimens were manufactured and tensile tested (3 iterations for each of the 14 possible combinations). In these tests, it was verified that all the specimens break transversely through the core and with a resistance greater than that specified by the supplier, which demonstrated the good performance of the different materials’ interfacial zones. These results demonstrate the feasibility of using combinations of these materials within the same component, with the associated advantages in terms of simplicity and reduction in the number of components.

In the second part of the work, the influence of the type of post-processing on the surface quality and tensile behavior was analyzed for several of the previously tested combinations. In view of the results achieved in the first part, the study was restricted to four combinations of Visijet CR-CL 200 with Visijet CE-NT, so that they were approximately equally spaced in terms of tensile strength: CE-NT, D60, D70, and CR-CL 200. In this way, the influence of three post-processing procedures could be assessed within the whole strength spectrum: furnace (F), ultrasonic with vegetable oil (OU), and ultrasonic with isopropyl alcohol (IU). Three iterations of the four combinations were used for each post-processing, in a total of 36 specimens. The results achieved in this part demonstrated the negative effect of using ultrasonics with isopropyl alcohol (IU) for the lower-strength specimens. This post-processing worsens the surface quality, limits the tensile strength, and reduces the deformation achievable by the material. In the case of the higher-strength specimens with lower CE-NT content, post-processing is less important for the elongation, but it has some influence on the strength. This behavior results from the detrimental effect of isopropyl alcohol on the CE-NT material. Therefore, for parts with a higher content of this material, post-processing in a furnace (F) or with ultrasonic and vegetable oil (OU) is recommended. For parts with a lower proportion of CE-NT, the furnace post-processing (F) stands out as the best option.

As future work, a study of all possible combinations of CR-CL 200 and CE-NT materials is proposed to carry out a more detailed characterization and, thus, to be able to identify in greater detail the combinations for which post-processing with isopropyl alcohol is detrimental. In addition, an in-depth analysis of the porosity of the multi-material parts using microscopy is proposed, so that the microscopic effect of the different post-processing on the material of the specimens can be studied. Thus, it will be possible to correlate the microscopic physical effect of the post-processing with the mechanical performance of the material in each case.

## Figures and Tables

**Table 1:** Main mechanical properties of resins and mixtures (according to ASTM D638) [9].

Material/Mixture	Tensile Strength (MPa)		Material/Mixture	Tensile Strength (MPa)
VisiJet CE-NT	0.20–0.40		D50	1–3
A40	0.23–0.32		D55	2–3
A50	0.35–0.48		D60	4–5
A60	0.48–0.77		D65	8–10
A70	0.75–1.10		D70	12–16
A80	1.30–1.70		D75	19–27
A90	1.40–1.90		VisiJet CR-CL 200	30–43

**Table 2:** Ra measured in the core of the specimens: individual values of the replicates and mean values.

		Ra (µm)			
Post-Process	Material/Mixture	CE-NT	D60	D70	CR-CL 200
F	Replica 1	2.9	0.8	2.7	2.4
	Replica 2	3.4	1.5	2.9	1.5
	Replica 3	3.0	1.6	3.2	1.6
	Mean	3.10	1.30	2.93	1.83
OU	Replica 1	1.9	1.6	2.2	1.5
	Replica 2	2.2	1.7	1.9	1.4
	Replica 3	2.0	1.6	2.1	1.3
	Mean	2.03	1.63	2.07	1.40
IU	Replica 1	8.9	1.6	1.7	1.5
	Replica 2	8.9	1.7	2.5	1.3
	Replica 3	8.8	3.0	1.6	1.3
	Mean	8.87	2.10	1.93	1.37

**Table 3:** P-values and correlations (*p*-value ≤ 0.05 criteria) between post-processing type and tensile performance ($\sigma_{R};\varepsilon \left(\sigma_{R}\right);\varepsilon \left(\sigma_{U}\right)$.

Material/Mixture	$\sigma_{R}$	$\varepsilon \left(\sigma_{R}\right)$	$\varepsilon \left(\sigma_{U}\right)$
CE-NT	0.000 (✓)	0.001 (✓)	0.001 (✓)
D60	0.048 (✓)	0.010 (✓)	0.054 (✕)
D70	0.031 (✓)	0.047 (✓)	0.132 (✕)
CR-CL 200	0.032 (✓)	0.110 (✕)	0.520 (✕)

## Data Availability

There are no shared data from this work.

## References

[1] Javaid M., Haleem A. (2018). Additive manufacturing applications in medical cases: A literature based review. Alex. J. Med..

[2] Najmon J.C., Raeisi S., Tovar A. (2019). Review of additive manufacturing technologies and applications in the aerospace industry. Additive Manufacturing for the Aerospace Industry.

[3] Nugraha A.D., Ruli, Supriyanto E., Rasgianti, Prawara B., Martides E., Junianto E., Wibowo A., Sentanuhady J., Muflikhun M.A. (2022). First-rate manufacturing process of primary air fan (PAF) coal power plant in Indonesia using laser powder bed fusion (LPBF) technology. J. Mater. Res. Technol..

[4] Nugraha A.D., Syahril M., Muflikhun M.A. (2023). Excellent performance of hybrid model manufactured via additive manufacturing process reinforced with GFRP for sport climbing equipment. Heliyon.

[5] Muflikhun M.A., Sentanu D.A. (2021). Characteristics and performance of carabiner remodeling using 3D printing with graded filler and different orientation methods. Eng. Fail. Anal..

[6] Patmonoaji A., Mahardika M.A., Nasir M., She Y., Wang W., Muflikhun M.A., Suekane T. (2022). Stereolithography 3D Printer for Micromodel Fabrications with Comprehensive Accuracy Evaluation by Using Microtomography. Geosciences.

[7] (1996). Additive Manufacturing General principles—Terminology.

[8] Tyagi S., Yadav A., Deshmukh S. (2022). Review on mechanical characterization of 3D printed parts created using material jetting process. Mater. Today Proc..

[9] VisiJet® Base Materials for the ProJet MJP 5600. https://es.3dsystems.com/multi-jet-printing.

[10] Stratasys PolyJet Technology. https://www.stratasys.com/es/polyjet-technology.

[11] Akbari S., Sakhaei A.H., Kowsari K., Yang B., Serjouei A., Yuanfang Z., Ge Q. (2018). Enhanced multimaterial 4D printing with active hinges. Smart Mater. Struct..

[12] Ge Q., Gu G., Wang D., Zhang B., Zhang N., Yuan C., Hingorani H., Ding N., Zhang Y.-F. (2019). Fast-response, stiffness-tunable soft actuator by hybrid multimaterial 3D printing. Adv. Funct. Mater..

[13] Dikshit V., Nagalingam P.A., Yap L.Y., Sing L.S., Yeong Y.W., Wei J. (2017). Investigation of quasi-static indentation response of inkjet printed sandwich structures under various indenter geometries. Materials.

[14] MultiJet Printing, Case of Study. https://es.3dsystems.com/motorsports/performance-wind-tunnel-testing?ind=motorsports.

[15] Davoudinejad A., Khosravani M.R., Pedersen D.B., Tosello G. (2020). Influence of thermal ageing on the fracture and lifetime of additively manufactured mold inserts. Eng. Fail. Anal..

[16] León-Cabezas M.A., Martínez-García A., Varela-Gandía F.J. (2017). Innovative advances in additive manufactured moulds for short plastic injection series. Procedia Manuf..

[17] Dämmer G., Gablenz S., Hildebrandt A., Major Z. Design and shape optimization of PolyJet bellows actuators. Proceedings of the 2018 IEEE International Conference on Soft Robotics (RoboSoft).

[18] Kakogawa A., Ma S.A. Differential Elastic Joint for Multi-linked Pipeline Inspection Robots. Proceedings of the 2018 IEEE/RSJ International Conference on Intelligent Robots and Systems (IROS).

[19] Pazhamannil R.V., Govindan P. (2021). Current state and future scope of additive manufacturing technologies via vat photopolymerization. Mater. Today Proc..

[20] Bezek L.B., Chatham C.A., Dillard D.A., Williams C.B. (2022). Mechanical properties of tissue-mimicking composites formed by material jetting additive manufacturing. J. Mech. Behav. Biomed. Mater..

[21] Khalid G.A., Bakhtiarydavijani H., Whittington W.R., Prabhu R., Jones M.D. (2020). Material response characterization of three poly jet printed materials used in a high fidelity human infant skull. Mater. Today Proc..

[22] Wang Y.C., Chen T., Yeh Y.L. (2019). Advanced 3D printing technologies for the aircraft industry: A fuzzy systematic approach for assessing the critical factors. Int. J. Adv. Manuf. Technol..

[23] Gao W., Zhang Y., Ramanujan D., Ramani K., Chen Y., Williams C.B., Wange C.C.L., Shin Y.C., Zhanga S., Zavattieri P.D. (2015). The status, challenges, and future of additive manufacturing in engineering. Comput. Aided Des..

[24] Yap Y.L., Wang C., Sing S.L., Dikshit V., Yeong W.Y., Wei J. (2017). Material jetting additive manufacturing: An experimental study using designed metrological benchmarks. Precis. Eng..

[25] Pugalendhi A., Ranganathan R., Ganesan S. (2021). Impact of process parameters on mechanical behaviour in multi-material jetting. Mater. Today Proc..

[26] Sagbas B., Gümüş B.E., Kahraman Y., Dowling D.P. (2021). Impact of print bed build location on the dimensional accuracy and surface quality of parts printed by multi jet fusion. J. Manuf. Process..

[27] Khoshkhoo A., Carrano A.L., Blersch D.M. (2018). Effect of surface slope and build orientation on surface finish and dimensional accuracy in material jetting processes. Procedia Manuf..

[28] Chen T., Dilag J., Bateman S. (2020). Surface topology modification of organic substrates using material jetting technologies. Mater. Des..

[29] Stanković T., Mueller J., Shea K. (2017). The effect of anisotropy on the optimization of additively manufactured lattice structures. Addit. Manuf..

[30] Sugavaneswaran M., Arumaikkannu G. (2015). Analytical and experimental investigation on elastic modulus of reinforced additive manufactured structure. Mater. Des..

[31] Abayazid F.F., Ghajari M. (2020). Material characterisation of additively manufactured elastomers at different strain rates and build orientations. Addit. Manuf..

[32] Liu W., Song H., Huang C. (2020). Maximizing mechanical properties and minimizing support material of PolyJet fabricated 3D lattice structures. Addit. Manuf..

[33] Meisel N.A., Williams C.B. Design for Additive Manufacturing: An investigation of key manufacturing considerations in multi-material PolyJet 3D printing. Proceedings of the 25th Annual International Solid Freeform Fabrication Symposium.

[34] Eren O., Sezer H.K., Yalçın N. (2022). Effect of lattice design on mechanical response of PolyJet additively manufactured cellular structures. J. Manuf. Process..

[35] He Y., Zhang F., Saleh E., Vaithilingam J., Aboulkhair N., Begines B., Tuck C.J., Hague R.J.M., Ashcroft I.A., Wildman R.D. (2017). A tripropylene glycol diacrylate-based polymeric support ink for material jetting. Addit. Manuf..

[36] Boopathy V.R., Sriraman A., Arumaikkannu G. (2019). Energy absorbing capability of additive manufactured multi-material honeycomb structure. Rapid Prototyp. J..

[37] (1996). Plastics Determination of Tensile Properties: Test Conditions for Moulding and Extrusion Plastics.

[38] Nguyen C.H.P., Kim Y., Choi Y. (2021). Design for additive manufacturing of functionally graded lattice structures: A design method with process induced anisotropy consideration. Int. J. Precis. Eng. Manuf.–Green. Technol..

[39] (1996). Geometrical Product Specifications (GPS)—Surface Texture: Profile Method—Rules and Procedures for the Assessment of Surface Texture.

[40] Bass L., Meisel N.A., Williams C.B. (2016). Exploring variability of orientation and aging effects in material properties of multi-material jetting parts. Rapid Prototyp. J..

